# Crohn’s Disease Remission with a Plant-Based Diet: A Case Report

**DOI:** 10.3390/nu11061385

**Published:** 2019-06-20

**Authors:** Kelsea Sandefur, Hana Kahleova, Alan N. Desmond, Eden Elfrink, Neal D. Barnard

**Affiliations:** 1 Kirksville College of Osteopathic Medicine, A.T. Still University, Kirksville, MO 12866, USA; eelfrink@atsu.edu; 2 Physicians Committee for Responsible Medicine, 5100 Wisconsin Ave, N.W. Ste.400, Washington, DC 20016, USA; hkahleova@pcrm.org (H.K.); nbarnard@pcrm.org (N.D.B.); 3 Devon Gut Clinic, Mount Stuart Hospital, Devon TQ1 7UP, UK; alan.desmond@nhs.net; 4 Department of Gastroenterology, South Devon Healthcare NHS Foundation Trust, Devon TQ2 7AA, UK; 5 Adjunct Faculty, George Washington University School of Medicine and Health Sciences, Washington, DC 20016, USA

**Keywords:** diet, nutrition, plant-based dietary lifestyle, whole food, Crohn’s disease, CD, inflammatory bowel disease, IBD, microbiome

## Abstract

Crohn’s disease (CD) is a form of chronic inflammatory bowel disease (IBD). The etiology of CD is thought to be multi-factorial; genetic factors, dietary and environmental exposures, immune events, and dysfunction of the gut microbiome are all though to play a role. The prevalence of CD is increasing globally and is higher in countries with a Westernized diet and lifestyle. Several human trials have demonstrated that plant-based dietary therapies may have utility in both the treatment of acute CD flares and the maintenance of remission. This case study describes a young adult male with newly diagnosed CD who failed to enter clinical remission despite standard medical therapy. After switching to a diet based exclusively on grains, legumes, vegetables, and fruits, he entered clinical remission without need for medication and showed no signs of CD on follow-up colonoscopy.

## 1. Introduction

Crohn’s disease (CD) was named for gastroenterologist Dr. Burrill B. Crohn who described a condition he called “terminal ileitis” in 1932 [1]. Descriptions of this condition can be found as far back as the 1700s [1]. The condition is commonly described as a disorder of uncertain etiology [2]. It is thought to be provoked by a combination of genetics, dietary and environmental exposures, immune changes, and an abnormal balance of gut bacteria [3].

CD is characterized by segments of transmural inflammation of the gastrointestinal tract which may occur anywhere from the mouth to the perianal area. Median age at diagnosis is 40 years [2]. Clinical manifestations are variable but include diarrhea, abdominal pain, rectal bleeding, fatigue, and weight loss. Segments of inflamed bowel are characterized by ulceration and polypoid mucosal changes which give a typical “cobblestone” appearance. Definitive diagnosis of CD is made by identifying the typical appearances at ileo-colonoscopy with biopsy and histological confirmation. Biopsies demonstrate typical granulomatous inflammation [2]. Up to one-third of patients have stricturing or penetrating intestinal complication at diagnosis, and half of all patients require surgery within 10 years of diagnosis [2]. Patients typically experience periods of inflammatory disease flares and periods of symptomatic remission. The majority of patients do not achieve prolonged clinical remission [3].

The prevalence of CD in the United States and Europe increased throughout the 20th century and has now plateaued. Approximately 1 in 315 people in the United States and 1 in 310 people in Germany have been diagnosed with CD [4]. In the early 21st century, CD has emerged as a global disease with accelerating incidence in newly industrialized countries [4]. Multiple epidemiological studies have found associations between dietary intakes and inflammatory bowel disease (IBD). Diets that are higher in refined carbohydrates, animal protein, total fat, and dairy fat have been positively associated with IBD prevalence [4,5,6,7,8,9,10]. IBD is also more common in populations with higher intakes of ultra-processed foods [11]. Conversely, higher intakes of fruits, vegetables, whole grains, and legumes may have a protective effect [7,10,11,12].

The gut microbiota may be a crucial mediator between diet and CD pathogenesis [13]. For example, common food emulsifiers and artificial flavor enhancers have been shown to promote the growth and pathogenic action of adherent-invasive *E. coli* (AIEC), harmful bacteria found in the microbiota of patients with CD [14,15]. In contrast, dietary concentrations of soluble plant polysaccharides have been shown to downregulate AIEC adherence [14]. A diet that is high in animal products and low in fiber can induce rapid changes in the human gut microbiota and provoke an outgrowth of bacterial subtypes that have been associated with IBD pathogenesis [16].

Prospective case series have shown that a diet that eliminates or restricts dairy foods, animal protein, animal fat, emulsifiers, and other artificial food additives can induce CD remission. This dietary intervention is effective in patients with newly diagnosed active CD and in patients with established CD who have failed to respond to standard medical therapy, with reported clinical remission rates of 78.7% and 90.4%, respectively [17,18]. Whole food dietary intervention is also well tolerated by patients with CD, with excellent adherence [19].

A diet that focuses on whole foods and plant-based sources of protein has also been reported to be beneficial in the maintenance of CD remission [20]. One controlled case series of 22 patients demonstrated a remission rate of 92% in CD patients who adhered to a whole food semi-vegetarian diet for two years, compared to 25% in patients who continued with a standard omnivorous diet.

Dietary intervention with exclusive enteral nutrition or elemental feeding is a well-established treatment for both adults and children with CD [21]. Recent advances in our understanding of CD pathogenesis suggest that this mode of treatment is successful because it excludes the common dietary components that may aggravate CD. A whole food dietary approach focused on plant-based sources of nutrition may be able to deliver the same therapeutic benefit [22].

## 2. Case

**Figure 1:**
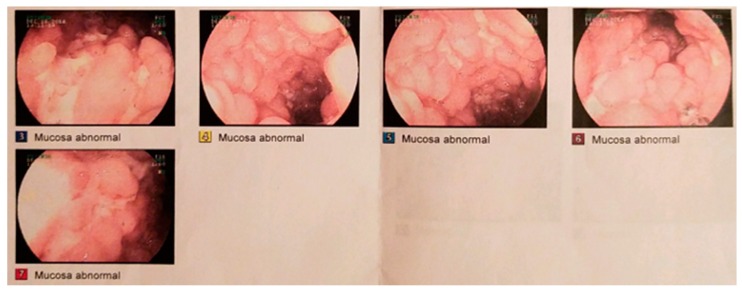
Photographs from diagnostic ileo-colonoscopy in December 2014: inflamed terminal ileum with the typical cobble stone mucosa and ulceration of Crohn’s disease.

In November 2014, a 25-year-old-male presented to the Department of Gastroenterology at a secondary care facility after having experienced several months of weight loss, diarrhea, and flu-like symptoms. He had a past medical history of peri-anal abscess and was on no prescribed or over-the-counter medications. He was a non-smoker. Ileo-colonoscopy demonstrated moderately inflamed mucosa with nodular congestion, marked erythema, and multiple shallow ulcers in the terminal ileum with minimal colonic involvement (Figure 1). The ileocecal valve appeared normal. A few small, non-bleeding mucosal erosions were present within the colon; at the hepatic flexure, transverse colon, and sigmoid colon. The overall impression was ileo-colonic Crohn’s disease with moderately severe and active ileal disease. Biopsies gave histopathological confirmation of moderately active ileal Crohn’s disease with mild, patchy colonic inflammation. At diagnosis, his symptoms indicated a Harvey-Bradshaw Index (HBI) score of 17, indicating moderately severe disease [23].

Treatment was commenced January 2015 with the biologic agent infliximab, 5 mg/kg by intravenous infusion every 8 weeks. After 37 weeks, the treatment dose was escalated to 7.5 mg/kg due to lack of symptomatic clinical response. The patient’s symptoms improved, but he did not achieve clinical remission. After 1 year of infliximab treatment, his HBI score was 5, indicating mildly active disease. Although he had demonstrated significant clinical response to therapy, he had failed to achieve clinical remission and continued to experience fatigue, bloating, and episodic severe abdominal pain.

**Figure 2:**
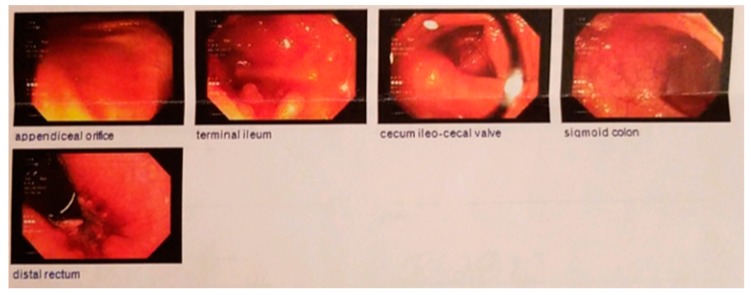
Follow-up ileo-colonoscopy August 2017: complete mucosal healing with no endoscopic evidence of Crohn’s disease.

In March 2017, having been on infliximab for 2 years, the patient reported a complete elimination of animal products and processed foods from his diet for forty days during a period of religious observation. During this time, he experienced a complete resolution of symptoms (HBI 0). Prior to this, his diet had been the typical American diet consisting of daily consumption of meat, dairy products, refined grains, processed foods, and modest amounts of vegetables and fruits. Having experienced complete clinical remission for the first time since his Crohn’s disease diagnosis, the patient decided to switch to a whole food, plant-based diet permanently, severely reducing his intake of processed foods and limiting animal products to one serving, or less, per week. There were a few reported periods of poor diet adherence, at which point symptoms of fatigue, nausea, bloating, and occasional aphthous ulcers would recur (HBI 2). HBI scores decreased back to 0 with adherence to the plant-based diet. He also began to employ stress-relief strategies including yoga, weight-lifting, and running. After 6 months of implementing these changes in diet and lifestyle, a follow-up ileo-colonoscopy in August 2017 demonstrated complete mucosal healing with no visible evidence of Crohn’s disease (Figure 2).

One year after this follow-up colonoscopy, infliximab therapy was ceased based upon ongoing complete clinical remission. As of May 2019, the patient had been off standard medical therapy for a total of 10 months and had not yet experienced a significant clinical relapse.

## 3. Discussion

Standard medical therapy for patients with newly diagnosed CD focuses on inducing and then maintaining remission. For low-risk patients, induction of remission is generally sought using tapering doses of oral budesonide or prednisolone [24], prescribed for durations of 6–8 weeks. Patients who require repeated courses of steroid medications are escalated to treatment with thiopurine medications (azathioprine or 6-mercaptopurine). If further treatment escalation is required, a biologic drug such as infliximab or adalimumab is commenced. In patients with high-risk features predictive of a more severe disease phenotype, clinicians will often employ a “top-down approach” with the early introduction of biologic drugs to induce and maintain remission. Maintenance therapy in high-risk patients requires ongoing therapy with thiopurines and/or biologic drugs for at least 12 months [25]. The patient in this case report was considered high-risk due to his moderately severe inflammation, severity of symptoms, diagnosis under the age of thirty, and his perianal disease. Despite current treatment regimens, only 10% of traditionally managed CD patients achieve long-term remission and 50% of patients require surgery within 10 years of diagnosis [25].

Both Kappelman et al. [26] and M’Koma [27] discussed the increasing global burden of IBD in recent years and the need for a plan on how to address this. Multiple human trials [16,17,18,19,20,21] have reported that a decrease in animal products and dairy and an increase in whole plant foods improved the clinical signs and symptoms of IBD patients, in particular CD. Chiba et al. [20] showed 100% maintenance of medically-induced remission at 2 years in 15 of 16 patients using a semi-vegetarian diet. For comparison, a combination of infliximab and azathioprine, without dietary intervention, has been reported to achieve 6-month clinical remission in only 57% of cases [28].

A whole food diet has previously been proposed as a successful adjunct to therapy in patients with CD. Case series of patients treated with a Crohn’s Disease Elimination Diet (CDED) have reported six-week remission rates of 70.6% in newly diagnosed patients and 62% in patients with established disease. The CDED protocol shares many of the defining characteristics of a whole food, plant-based diet: excluding or minimizing exposure to food emulsifiers, flavor enhancers, sources of omega-6 fatty acids, and dairy products, while providing sources of plant poly-saccharides and dietary fiber [18].

A plant-based diet appears to be beneficial for human health by promoting the development of more diverse microbial systems [13]. Higher fiber intake also encourages the growth of species that ferment fiber into metabolites as short-chain fatty acids (SCFAs), including acetate, propionate, and butyrate. The positive health effects of SCFAs are myriad, including improved immunity against pathogens, blood–brain barrier integrity, provision of energy substrates, and regulation of critical functions of the intestine [29].

Two limitations of this case report are that the patient was on therapy during the duration in which the dietary alteration occurred and that the diet was not recorded with a food diary. A diet consisting of whole grains, legumes, fruits, and vegetables has been shown to be helpful in the prevention and treatment of heart disease, obesity, diabetes, hypertension, gallbladder disease, rheumatoid arthritis, and many cancers [30]. Although further research is required, taken in the context of emerging evidence, this case report suggests that Crohn’s disease might be added to this list of conditions.
